# The optimal time interval between neoadjuvant chemoradiotherapy and surgery for patients with an unfavorable pathological response in locally advanced rectal cancer: a retrospective cohort study

**DOI:** 10.3389/fonc.2025.1534148

**Published:** 2025-02-14

**Authors:** Litao Wang, Jianyong Fan, Yaqi Guo, Shipeng Shang, Han Gao, Jianfei Xu, Peng Gao, Enrui Liu

**Affiliations:** ^1^ Department of Emergency Surgery, The Affiliated Hospital of Qingdao University, Qingdao University, Qingdao, Shandong, China; ^2^ Department of Emergency Medicine, The Affiliated Hospital of Qingdao University, Qingdao University, Qingdao, Shandong, China; ^3^ Department of Anesthesiology, The Affiliated Hospital of Qingdao University, Qingdao University, Qingdao, Shandong, China; ^4^ Clinical Research Center (CRC), Medical Pathology Center (MPC), Cancer Early Detection and Treatment Center (CEDTC), Chongqing University Three Gorges Hospital, Chongqing University, Chongqing, China; ^5^ Translational Medicine Research Center (TMRC), School of Medicine Chongqing University, Chongqing, China; ^6^ School of Basic Medicine, Qingdao University, Qingdao, Shandong, China

**Keywords:** locally advanced rectal cancer, neoadjuvant chemoradiation, tumor response grading, unfavorable pathological response, surgical interval

## Abstract

**Background:**

The focus of this study was to determine the optimal time interval between neoadjuvant chemoradiotherapy (nCRT) and surgery in patients with locally advanced rectal cancer (LARC) who had an unfavorable pathological response, as well as to investigate the correlation between long-term outcomes and the duration of this interval.

**Methods:**

The present study retrospectively analyzed patients with locally advanced rectal cancer who underwent nCRT followed by total mesorectal excision between (TME) January 2018 and September 2021. Patients included in this study had an unfavorable pathological response, confirmed as tumor regression grade (TRG) 2-3. X-tile analysis was subsequently conducted to determine the optimal cut-off value for the time interval between nCRT and surgery. Furthermore, Cox proportional hazards regression analyses were performed to identify independent prognostic factors, and the Kaplan-Meier method was used to estimate long-term survival.

**Results:**

The study cohort comprised of 114 patients (51.35%) in the longer interval group (>8 weeks), while the remaining 108 patients (48.65%) belonged to the shorter interval group (≤8 weeks). Univariable and multivariate Cox proportional hazards regression analyses revealed that a longer interval time was identified as an independent risk factor for overall survival (HR: 2.14, 95% CI: 1.01-4.55, *P*=0.048) and disease-free survival (HR: 2.03, 95% CI: 1.09-3.77, *P*=0.025) among these patients. Moreover, patients in the longer interval group exhibited significantly worse OS and DFS compared to those in the shorter interval group (3-year OS: 87.2% vs 68.2%, *P*=0.001; 3-year DFS: 80.4% vs 62.7%, *P*=0.003). Furthermore, similar results were observed in subgroup analyses based on different TRG scores.

**Conclusions:**

The surveillance and monitoring should be promptly conducted following nCRT in order to promptly identify patients with an unfavorable pathological response, who would benefit from timely radical surgery within 8 weeks.

## 1 Introduction

Neoadjuvant chemoradiotherapy and total mesorectal excision are currently recommended as the established standard treatment for locally advanced rectal cancer (1, 2). The implementation of this standardized approach leads to improved long-term outcomes and higher rates of sphincter preservation compared to postoperative chemoradiotherapy alone. Consequently, a greater number of patients can maintain normal bowel function without the need for permanent colostomy bags (3–6).

The timing of surgery is crucial for patients who have undergone neoadjuvant chemoradiotherapy due to the time-dependent impact of ionizing radiation on tumors (7, 8). The duration between nCRT and surgery plays a pivotal role in promoting tumor regression and downstaging (5, 9). However, an extended interval may result in therapy-induced pelvic fibrosis and anatomical difficulties, leading to increased surgical complications (10, 11). While only a minority of patients achieve a pathological complete response (pCR) following neoadjuvant chemoradiotherapy, allowing for the implementation of a “watch-and-wait” strategy while disregarding surgical complications, the majority experience an unfavorable pathological response, making it challenging to determine the optimal time interval between nCRT and surgery (12–15).

The aim of this study was to determine the optimal time interval between neoadjuvant chemoradiotherapy and surgery in patients with locally advanced rectal cancer who had an unfavorable pathological response, as well as to investigate the correlation between long-term outcomes and the duration of this interval.

## 2 Materials and methods

### 2.1 Data source

The study cohort consisted of patients who underwent neoadjuvant therapy and radical resection for locally advanced rectal cancer at the affiliated hospital of Qingdao University between January 2018 and September 2021. The study protocol was approved by the ethics committee of the affiliated hospital of Qingdao University.

### 2.2 Study population

Patients included in this study were required to meet the following criteria: (1) The diagnosis of rectal adenocarcinoma in patients was confirmed through pathological examination; (2) The patients underwent pretreatment rectal MRI and were classified as having LARC; (3) The patients underwent nCRT followed by standard TME surgery; (4) The pathological response of the patients was confirmed to be TRG 2-3; (5) The patients diagnosed after reaching the age of 18 years old.

The exclusion criteria were as follows: (1) The pathological examination confirmed alternative histological categorizations; (2) The patients were accompanied by the presence of distant metastasis; (3) The patients did not receive the standard nCRT and TME surgery; (4) The patients who have been lost to follow-up.

### 2.3 Pathological response

The histopathologic analysis was independently reported by two pathologists. The TRG was conducted based on the American Joint Committee on Cancer TRG System, which consists of four categories ranging from 0 to 3 (16). Each category represents the degree of cancer cell eradication.

- TRG 0 represents complete regression without any remaining cancer cells.
- TRG 1 indicates near-complete regression with only one isolated residual cancer cell or a cluster of cancer cells.
- TRG 2 signifies moderate regression with numerous residual cancer cells still present.
- TRG 3 suggests minimal regression where almost no cancer cells have been eradicated.

### 2.4 Neoadjuvant chemoradiotherapy regimens

For patients undergoing long-term radiotherapy, pelvic radiation is generally delivered at a dose of 45-50.4 Gy in 25 fractions, concurrently administered with capecitabine (825 mg/m², orally, twice daily on weekdays). Short-term radiotherapy (25 Gy in 5 fractions) is indicated for rectal cancer patients with MRI-staged T3 tumors that do not require sphincter preservation. Upon completion of radiation therapy, patients undergo consolidation chemotherapy, which provides two treatment options: (1) Monotherapy with oral capecitabine administered at a dose of 1250 mg/m² twice daily for 14 days, repeated every 3 weeks. (2) The CapeOx regimen, consisting of oxaliplatin (130 mg/m² on day 1) combined with capecitabine (1000 mg/m² orally, twice daily from day 1 to day 14), with cycles repeated every 3 weeks.

### 2.5 Follow-up

Patients generally schedule their first postoperative follow-up appointment 4 to 6 weeks after surgery. Following the initial treatment, patients undergo monitoring every 3 months during the first 3 years, every 6 months during years 4 to 5, and annually thereafter. The follow-up plan encompasses a comprehensive series of evaluations, including clinical examinations, assessments of tumor marker, colonoscopy, CT scans of the chest and abdomen, and pelvic CT or MRI. Survival data were collected via comprehensive medical record reviews and structured telephone follow-ups, with the follow-up period concluding on June 30, 2024. Overall survival (OS) is defined as the interval from the date of surgery to the date of death. Disease-free survival (DFS) is defined as the interval from the date of surgery to the first occurrence of local recurrence, distant metastasis, or death.

### 2.6 Statistical analysis

All statistical analyses were conducted with R software version 3.4.0 (http://www.R-project.org) and all graphics were performed with GraphPad Prism (version 10.3.1). The Chi-squared test was used to compare proportions. And Univariable and multivariate Cox proportional hazards regression analyses were performed to identify the independent prognostic factors. Kaplan-Meier method estimated the survival curves using the log-rank test. The statistical tests conducted were two-sided, and significance was determined at a threshold of P values <0.05.

## 3 Results

### 3.1 Clinical characteristics

**Figure 1:**
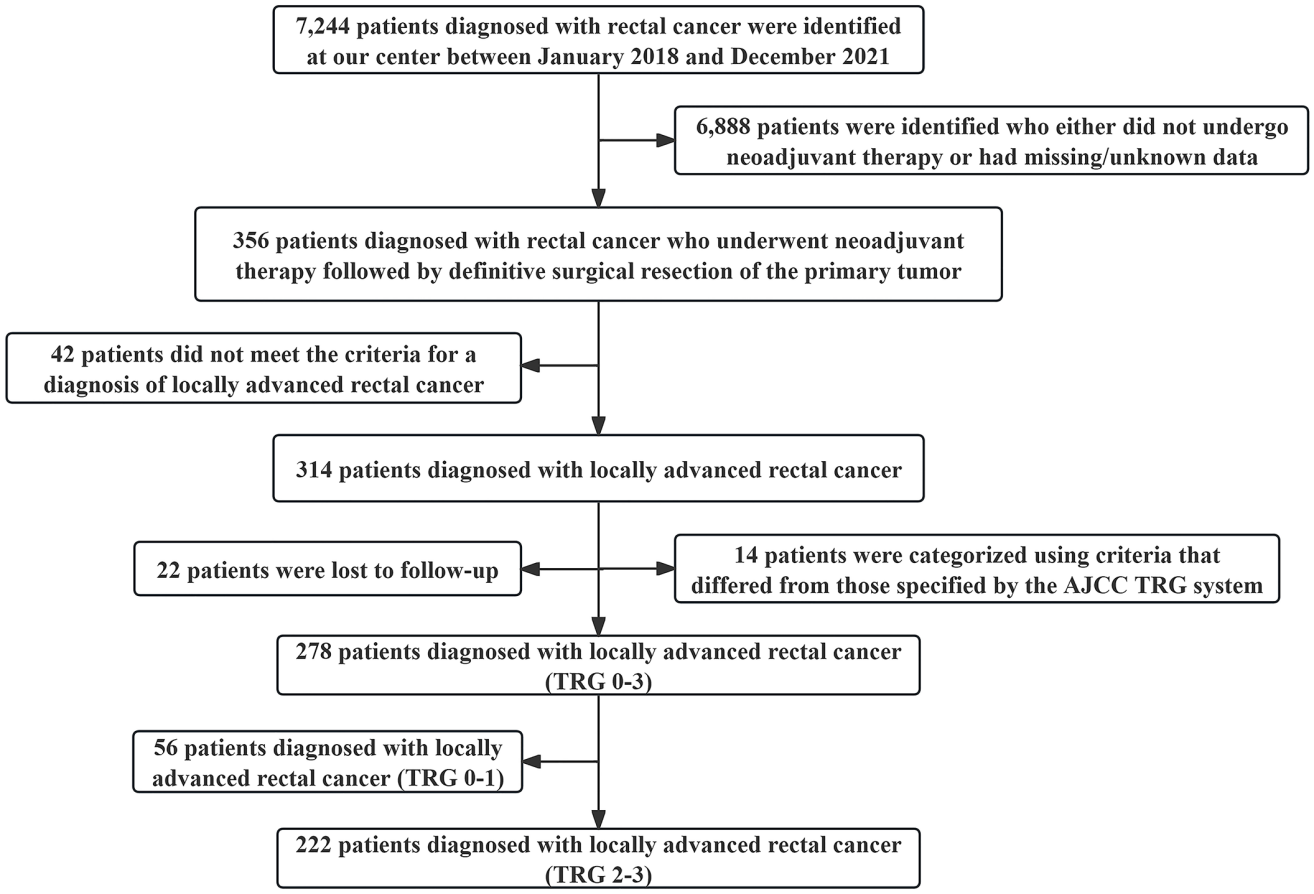
The study flowchart of the patient selection process.

A total of 278 eligible patients with locally advanced rectal cancer were enrolled in this study, all of whom underwent standard neoadjuvant chemoradiotherapy followed by total mesorectal excision surgery (**Figure 1**). Among them, 56 patients who achieved a favorable response (TRG 0-1) were excluded from this study, while the remaining 222 patients who had an unfavorable response (TRG 2-3) were included. Subsequently, X-tile analysis was conducted to determine the optimal cut-off value for the time interval between neoadjuvant chemoradiotherapy and surgery, which was found to be 54 days (approximately 8 weeks) in **Supplementary Figure 1**. The study cohort consisted of 114 out of the 222 patients (51.35%) in the longer interval group (>8 weeks), whereas the remaining 108 out of the 222 patients (48.65%) belonged to the shorter interval group (≤8 weeks) (**Table 1**).

**Table 1:** Demographical characteristics of patients.

Variable	Interval			
	Total	≤8 weeks	>8 weeks	*p*
All patients	222	114	108	
Age (years)				0.051
≤50	65 (29.28)	40 (35.09)	25 (23.15)	
>50	157 (70.72)	74 (64.91)	83 (76.85)	
Sex				0.421
Male	161 (72.52)	80 (70.18)	81 (75.00)	
Female	61 (27.48)	34 (29.82)	27 (25.00)	
BMI				0.919
<24	102 (45.95)	52 (45.61)	50 (46.30)	
≥24	120 (54.05)	62 (54.39)	58 (53.70)	
Differentiation				0.041
Poor	32 (14.41)	15 (13.16)	17 (15.74)	
Moderate	161 (72.52)	90 (78.95)	71 (65.74)	
Well	29 (13.06)	9 (7.89)	20 (18.52)	
Preop-CEA				0.127
Normal	122 (54.95)	57 (50.00)	65 (60.19)	
Abnormal	100 (45.05)	57 (50.00)	43 (39.81)	
Preop-CA199				0.909
Normal	174 (78.38)	89 (78.07)	85 (78.70)	
Abnormal	48 (21.62)	25 (21.93)	23 (21.30)	
Surgical approach				0.088
Miles	51 (22.97)	25 (21.93)	26 (24.07)	
Dixon	146 (65.77)	81 (71.05)	65 (60.19)	
Hartmann	25 (11.26)	8 (7.02)	17 (15.74)	
Chemotherapy				0.117
capecitabine	60 (27.03)	36 (31.58)	24 (22.22)	
CapeOx	162 (72.97)	78 (68.42)	84(77.78)	
Radiotherapy				0.541
long-term RT	204 (91.89)	106 (92.98)	98 (90.74)	
short-term RT	18 (8.11)	8 (7.02)	10 (9.26)	
cT stage				0.169
T1	22 (9.91)	12 (10.53)	10 (9.26)	
T2	31 (13.96)	18 (15.79)	13 (12.04)	
T3	139 (62.61)	64 (56.14)	75 (69.44)	
T4	30 (13.51)	20 (17.54)	10 (9.26)	
cN stage				0.001
N0	62 (27.93)	37 (32.46)	25 (23.15)	
N1	77 (34.68)	49 (42.98)	28 (25.93)	
N2	83 (37.39)	28 (24.56)	55 (50.93)	
CRM				0.641
Negative	105 (47.30)	61 (53.51)	56 (51.85)	
Positive	117 (52.70)	53 (46.49)	52 (48.15)	
ypT stage				0.130
T1	35 (15.77)	20 (17.54)	15 (13.89)	
T2	64 (28.83)	29 (25.44)	35 (32.41)	
T3	111 (50.00)	62 (54.39)	49 (45.37)	
T4	12 (5.41)	3 (2.63)	9 (8.33)	
ypN stage				0.006
N0	95 (42.79)	60 (52.63)	35 (32.41)	
N1	76 (34.23)	35 (30.70)	41 (37.96)	
N2	51 (22.97)	19 (16.67)	32 (29.63)	
Perineural invasion				0.033
Negative	106 (47.75)	64 (56.14)	42 (38.89)	
Positive	62 (27.93)	28 (24.56)	34 (31.48)	
Unknown	54 (24.32)	22 (19.30)	32 (29.63)	
Vascular invasion				0.032
Negative	137 (61.71)	79 (69.30)	58 (53.70)	
Positive	40 (18.02)	14 (12.28)	26 (24.07)	
Unknown	45 (20.27)	21 (18.42)	24 (22.22)	
Tumor location (cm)				0.774
0-5	76 (34.23)	41 (35.96)	35 (32.41)	
6-10	122 (54.95)	60 (52.63)	62 (57.41)	
11-15	24 (10.81)	13 (11.40)	11 (10.19)	
Tumor size (cm)				0.923
≤3	137 (61.71)	70 (61.40)	67 (62.04)	
>3	85 (38.29)	44 (38.60)	41 (37.96)	
TRG				0.940
2	167 (75.23)	86 (75.44)	81 (75.00)	
3	55 (24.77)	28 (24.56)	27 (25.00)	

CRM, circumferential resection margin; BMI, Body Mass Index; TRG, Tumor Regression Grade.

### 3.2 Univariable and multivariate Cox regression analyses

Univariable and multivariate Cox proportional hazards regression analyses were conducted to assess the impact of the interval time from nCRT to surgery on OS in patients with an unfavorable pathological response in locally advanced rectal cancer. The results indicated that longer interval time was identified as an independent risk factor for OS (HR: 2.14, 95% CI: 1.01-4.55, *P*=0.048) among these patients (**Table 2**). Tumor differentiation, vascular invasion, TRG, and interval time were considered potential confounders.

**Table 2:** Univariate and multivariate Cox proportional hazards regression analyses for overall survival.

Variable	Univariable analysis			Multivariable analysis		
	HR	95% Cl	*P*	HR	95% Cl	*P*
Age (years)						
≤50	1.00					
>50	1.83	0.85 ~ 3.95	**0.125**			
Sex						
Female	1.00			1.00		
Male	0.50	0.27 ~ 0.92	**0.026**	0.70	0.33 ~1.46	**0.341**
BMI						
<24	1.00					
≥24	1.17	0.63 ~ 2.15	**0.621**			
Differentiation						
Poor	1.00					
Moderate	0.38	0.18 ~ 0.80	**0.011**	0.40	0.16~0.98	**0.045**
Well	1.23	0.52 ~ 2.90	**0.635**	1.25	0.47~3.30	**0.652**
Preop-CEA						
Normal	1.00					
Abnormal	1.13	0.62 ~ 2.07	**0.688**			
Preop-CA199						
Normal	1.00					
Abnormal	1.43	0.72 ~ 2.84	**0.310**			
Surgical approach						
Miles	1.00			1.00		
Dixon	0.70	0.32 ~ 1.54	**0.371**	0.80	0.35 ~ 1.85	**0.605**
Hartmann	4.08	1.77 ~ 9.44	**<0.001**	2.45	0.96 ~ 6.29	**0.062**
Chemotherapy						
Capecitabine	1.00			1.00		
CapeOx	0.47	0.25 ~ 0.86	**0.015**	0.63	0.30 ~1.33	**0.227**
Radiotherapy						
long-term RT	1.00					
short-term RT	0.23	0.03 ~ 1.66	**0.144**			
cT stage						
T1	1.00					
T2	4.39	0.51 ~ 37.59	**0.177**			
T3	6.35	0.86 ~ 46.59	**0.060**			
T4	6.09	0.73 ~ 50.73	**0.095**			
cN stage						
N0	1.00					
N1	0.48	0.19 ~ 1.19	**0.111**			
N2	1.47	0.72 ~ 3.03	**0.291**			
CRM						
Negative	1.00					
Positive	0.73	0.39 ~ 1.35	**0.311**			
ypT stage						
T1	1.00					
T2	1.04	0.38 ~ 2.81	**0.940**			
T3	1.29	0.52 ~ 3.21	**0.581**			
T4	2.90	0.82 ~ 10.29	**0.099**			
ypN stage						
N0	1.00			1.00		
N1	0.90	0.38 ~ 2.14	**0.817**	0.60	0.24 ~ 1.53	**0.288**
N2	3.83	1.89 ~ 7.79	**<0.001**	2.21	1.02 ~ 4.79	**0.044**
Perineural invasion						
Negative	1.00					
Positive	2.69	0.57 ~ 3.05	**0.005**	1.63	0.72 ~ 3.67	**0.240**
Unknown	1.32	0.57 ~ 3.05	**0.515**	0.77	0.21 ~ 2.74	**0.682**
Vascular invasion						
Negative	1.00			1.00		
Positive	4.00	2.11 ~ 7.57	**<0.001**	2.84	1.34 ~ 5.99	**0.006**
Unknown	0.60	0.21 ~ 1.76	**0.355**	1.22	0.23 ~ 6.38	**0.816**
TRG						
2	1.00			1.00		
3	3.02	1.65 ~ 5.53	**<0.001**	2.17	1.13 ~ 4.13	**0.019**
Tumor location (cm)						
0-5	1.00					
6-10	1.23	0.61 ~ 2.48	**0.556**			
11-15	2.15	0.85 ~ 5.47	**0.107**			
Tumor size (cm)						
≤3	1.00					
>3	0.85	0.45 ~ 1.61	**0.624**			
Interval						
≤8 weeks			1.00			1.00
>8 weeks	2.92	1.47~ 5.80	**0.002**	2.14	1.01 ~ 4.55	**0.048**

HR, hazard ratio; CI, confidence interval; CRM, circumferential resection margin; BMI, Body Mass Index; TRG, Tumor Regression Grade.

P values<0.05 were considered as the significance threshold.

For DFS, Cox proportional hazards regression analyses were performed to evaluate the association between the interval time from nCRT to surgery and DFS in patients with an unfavorable pathological response in locally advanced rectal cancer. The findings revealed that longer interval time was independently associated with a higher risk of DFS events (HR: 2.03, 95% CI: 1.09-3.77, *P*=0.025) among these patients (**Table 3**). Tumor differentiation and preoperative cancer antigen 199 (Preop-CA199) were included as potential confounders.

**Table 3:** Univariate and multivariate Cox proportional hazards regression analyses for disease-free survival.

Variable	Univariable analysis			Multivariable analysis		
	HR	95% Cl	*P*	HR	95% Cl	*P*
Age (years)						
≤50	1.00					
>50	1.14	0.63~2.06	**0.667**			
Sex						
Female	1.00					
Male	0.68	0.39 ~ 1.18	**0.166**			
BMI						
<24	1.00					
≥24	1.26	0.74 ~ 2.13	**0.399**			
Differentiation						
Poor	1.00					
Moderate	0.36	0.19 ~ 0.67	**0.001**	0.50	0.25~0.99	**0.048**
Well	0.89	0.41 ~ 1.93	**0.769**	1.16	0.48~2.76	**0.745**
Preop-CEA						
Normal	1.00					
Abnormal	1.33	0.79 ~ 2.24	**0.292**			
Preop-CA199						
Normal	1.00					
Abnormal	3.18	1.86 ~ 5.44	**<0.001**	3.18	1.75 ~5.77	**<0.001**
Surgical approach						
Miles	1.00					
Dixon	1.12	0.55 ~ 2.28	**0.745**	1.30	0.56 ~ 2.99	**0.538**
Hartmann	3.27	1.43 ~ 7.47	**0.005**	1.61	0.62 ~ 4.18	**0.327**
Chemotherapy						
Capecitabine	1.00					
CapeOx	0.71	0.41 ~ 1.24	**0.228**			
Radiotherapy						
long-term RT	1.00					
short-term RT	0.39	0.10~ 1.61	**0.194**			
cT stage						
T1	1.00					
T2	0.93	0.21 ~ 4.15	**0.924**			
T3	2.45	0.76 ~ 7.93	**0.134**			
T4	2.32	0.61 ~ 8.76	**0.214**			
cN stage						
N0	1.00					
N1	0.55	0.26 ~ 1.17	**0.121**			
N2	1.46	0.78 ~ 2.73	**0.232**			
CRM						
Negative	1.00					
Positive	0.81	0.48 ~ 1.38	**0.448**			
ypT stage						
T1	1.00					
T2	0.61	0.25 ~ 1.47	**0.269**			
T3	1.16	0.55 ~ 2.45	**0.689**			
T4	1.97	0.66 ~ 5.88	**0.224**			
ypN stage						
N0	1.00			1.00		
N1	0.83	0.41 ~ 1.68	**0.600**	0.84	0.41 ~ 1.75	**0.647**
N2	2.71	1.48 ~ 4.95	**0.001**	1.96	1.00 ~ 3.84	**0.051**
Perineural invasion						
Negative	1.00					
Positive	1.91	1.05 ~ 3.44	**0.033**	1.35	0.69 ~ 2.63	**0.377**
Unknown	1.04	0.51 ~ 2.09	**0.922**	1.50	0.48 ~ 4.70	**0.482**
Vascular invasion						
Negative	1.00			1.00		
Positive	2.64	1.48 ~ 4.70	**<0.001**	1.55	0.75 ~ 3.19	**0.236**
Unknown	0.67	0.29 ~ 1.52	**0.335**	0.59	0.15 ~ 2.26	**0.438**
TRG						
2	1.00			1.00		
3	2.16	1.27 ~ 3.68	**0.005**	1.70	0.95 ~ 3.03	**0.074**
Tumor location (cm)						
0-5	1.00					
6-10	1.57	0.61 ~ 2.48	**0.159**	1.28	0.62 ~ 2.65	**0.507**
11-15	2.36	1.02 ~ 5.46	**0.045**	1.92	0.74 ~ 5.01	**0.183**
Tumor size (cm)						
≤3	1.00					
>3	0.91	0.53 ~ 1.58	**0.747**			
Interval						
≤8 weeks	1.00			1.00		
>8 weeks	2.26	1.29 ~ 3.96	**0.004**	2.03	1.09 ~ 3.77	**0.025**

HR, hazard ratio; CI, confidence interval; CRM, circumferential resection margin; BMI, Body Mass Index; TRG, Tumor Regression Grade.

P values<0.05 were considered as the significance threshold.

### 3.3 The correlation between the time interval and survival outcomes

**Figure 2:**
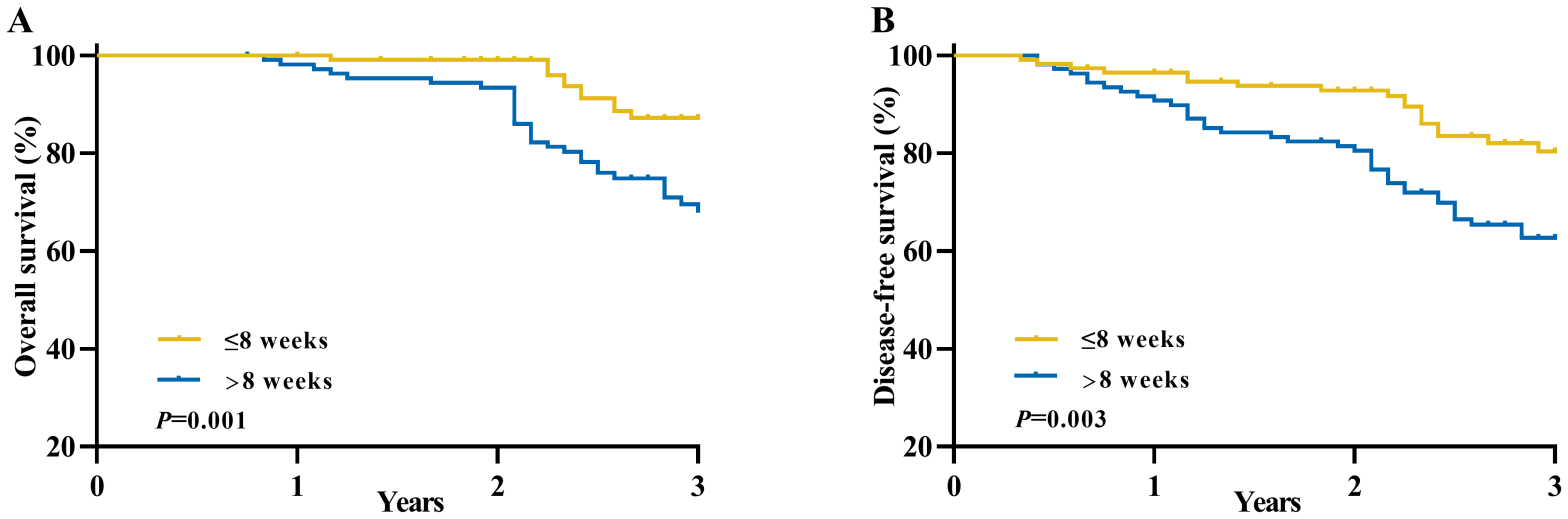
Long-term survival of patients with an unfavorable pathological response in locally advanced rectal cancer between the longer interval and shorter interval groups. **(A)** Overall survival and **(B)** Disease-free survival.

The median follow-up duration in this study was 34 months (**Supplementary Table 1**), during which a total of 56 patients (25.2%) experienced local recurrences or distant metastases, and 42 patients (18.9%) succumbed to mortality. The Kaplan-Meier method was utilized to estimate the long-term OS and DFS rates in patients diagnosed with locally advanced rectal cancer. And patients in the longer interval group exhibited significantly worse OS and DFS rates at 3 years compared to the shorter interval group (3-year OS: 87.2% vs. 68.2%, *P*= 0.001; 3-year DFS: 80.4% vs. 62.7%, *P*=0.003) (**Figure 2**).

### 3.4 Survival outcomes by different TRG scores

**Figure 3:**
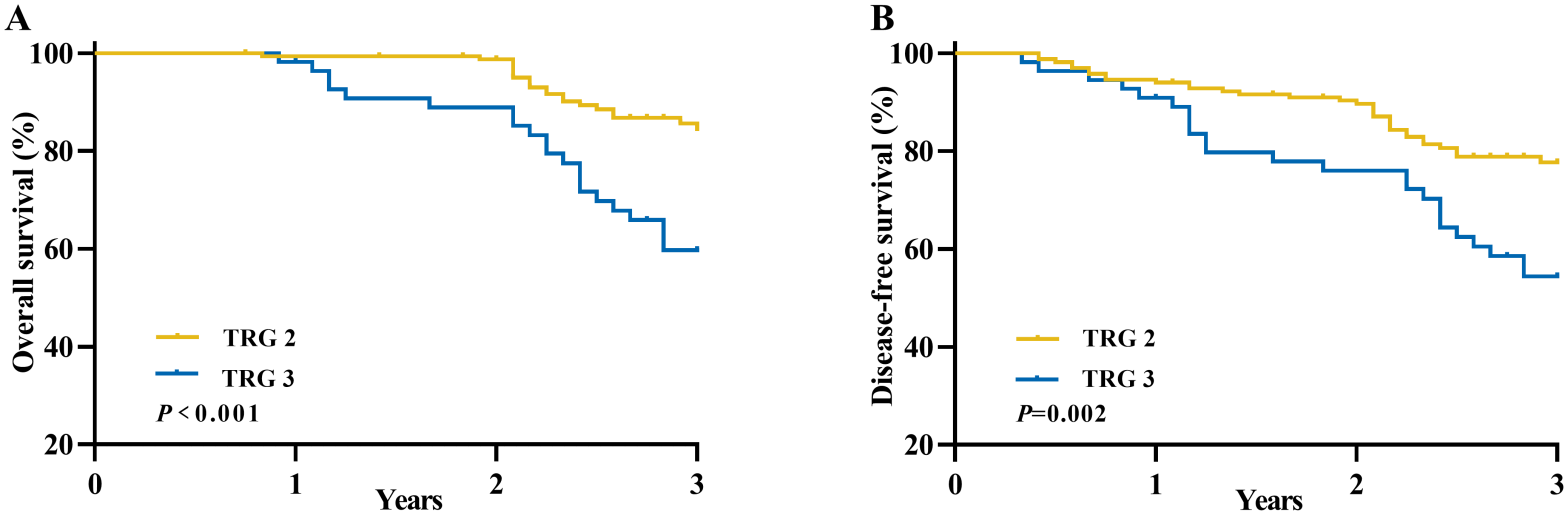
Long-term survival of patients with an unfavorable pathological response in locally advanced rectal cancer according to TRG scores. **(A)** Overall survival and **(B)** Disease-free survival.

The Kaplan-Meier method was utilized to compare the survival outcomes based on different TRG scores in **Figure 3**, revealing that patients with a TRG 2 score demonstrated superior OS (3-years OS: 84.5% vs. 59.7%, P< 0.001) and DFS (3-years DFS: 77.7% vs. 54.4%, *P*=0.002) in comparison to those with a TRG 3 score.

**Figure 4:**
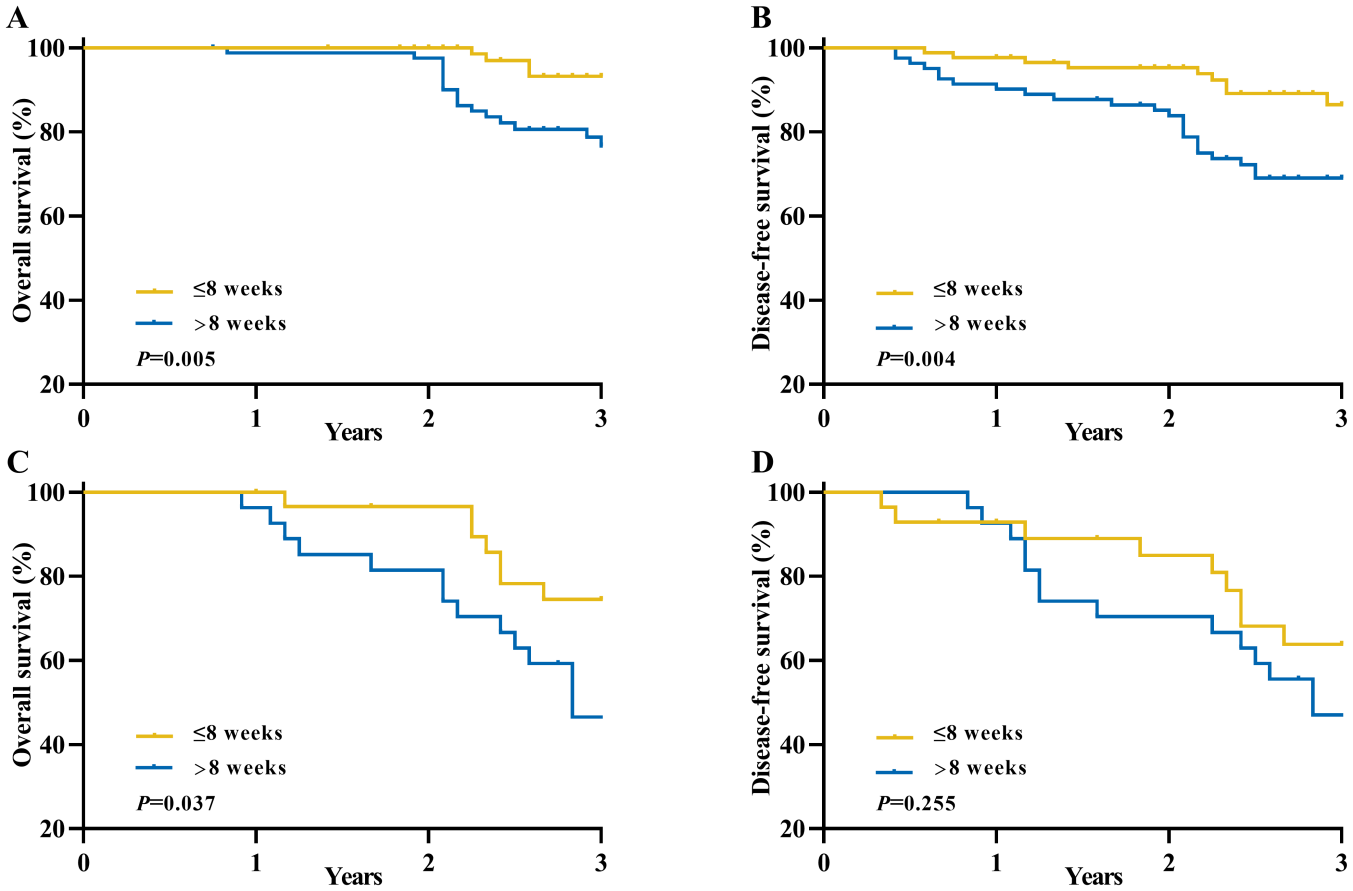
Long-term survival of patients with an unfavorable pathological response in locally advanced rectal cancer between the longer interval and shorter interval groups according to TRG scores. **(A)** Overall survival for TRG 2 group; **(B)** Disease-free survival for TRG 2 group; **(C)** Overall survival for TRG 3 group; **(D)** Disease-free survival for TRG 3 group.

Furthermore, we conducted additional subgroup analyses to further investigate the association between the time interval and survival outcomes among different TRG scores groups (**Figure 4**). The results of these subgroup analyses were consistent with our previous findings, demonstrating that patients in the longer interval group had significantly worse OS (3-year OS: 93.2% vs. 76.7%, *P=*0.005) and DFS (3-year DFS: 86.4% vs. 69.1%, *P=*0.004) compared to those in the shorter interval group within the TRG 2 category. Similar trends were observed within the TRG 3 category for OS (3-year OS: 74.5% vs. 46.6%, *P*=0.037) and DFS (3-year DFS: 63.9%vs. 47.0%, *P*=0.255).

### 3.5 Survival outcomes by different nCRT treatment strategies

To explore the impact of different nCRT treatment strategies on patient prognosis, we conducted a subgroup analysis of various radiotherapy and chemotherapy regimens. In the chemotherapy subgroup analysis (**Supplementary Figure 2**), patients in the capecitabine group with a longer interval had significantly worse OS and DFS compared to those with shorter interval (3-year OS: 79.6% vs. 39.8%, *P*=0.004; 3-year DFS: 78.2% vs. 43.3%, *P*=0.011). In the CapeOx group, the longer interval group also exhibited worse prognosis (3-year OS: 90.7% vs. 77.2%, *P*=0.025; 3-year DFS: 81.3% vs. 69.2%, *P*=0.049). In the radiotherapy subgroup analysis (**Supplementary Figure 3**), patients in the long-term radiotherapy group showed a similar trend. Specifically, the OS and DFS in the longer interval group were significantly lower than those in the shorter interval group (3-year OS: 86.2% vs. 66.3%, *P*=0.001; 3-year DFS: 79.6% vs. 63.4%, *P*=0.010). However, in the short-term radiotherapy group, no statistically significant differences in OS and DFS were observed between the long and short interval groups, likely due to sample size limitations.

## 4 Discussion

To enhance tumor downstaging and increase the rate of sphincter preservation, the National Comprehensive Cancer Network (NCCN) guidelines recommend administering preoperative neoadjuvant therapy to patients diagnosed with locally advanced rectal cancer (17). Neoadjuvant therapy involves the administration of chemotherapy or radiation therapy prior to surgery in order to reduce tumor size and potentially render them more amenable for surgical intervention, thereby obviating the need for permanent colostomy. However, the optimal timing for interval between neoadjuvant chemoradiotherapy and surgery in patients with locally advanced rectal cancer remains a subject of ongoing research and debate (18–20). This study meticulously collected the precise interval between neoadjuvant therapy and surgery in patients with an unfavorable pathological response, determining that an inflection point for OS and DFS occurs at approximately the 8th week. The OS and DFS were compared between the longer interval group (>8 weeks) and the shorter interval group (≤8 weeks). Importantly, the findings demonstrated that patients in the shorter interval group exhibited more favorable outcomes, indicating that a reduced time period following nCRT may have contributed to improved OS and DFS.

The presence of a pathological response is correlated with patient survival, and a favorable response indicates an improved long-term prognosis (21–23). The prolongation of the interval time often signifies a more favorable pathologic response and higher probability of achieving complete pathological response, thereby necessitating additional time for tumor regression and downstaging (14, 24, 25). However, the option of extended interval time seems to confer benefits for patients demonstrating a favorable pathological response, while it does not appear to improve the oncological outcome for patients with an unfavorable pathological response (11). Research findings indicate that the rate of tumor shrinkage gradually diminishes over time, with the most rapid reduction occurring during the initial stages of nCRT and the slowest just prior to surgery (18). So the extension of the interval duration may potentially contribute to tumor progression and distant metastasis in patients exhibiting an unfavorable pathological response, ultimately impacting long-term survival outcomes negatively (10). The present study unveiled that individual in the longer interval group, who exhibited an unfavorable pathological response, demonstrated significantly inferior rates of OS and DFS compared to those in the shorter interval group. Therefore, it is crucial to consider that timely intervention plays a significant role in managing cancer effectively. By minimizing the time intervals between nCRT and surgery, we have the potential to mitigate the likelihood of tumor progression and distant metastasis. And the previous study demonstrated similar findings, indicating that patients in the longer interval group had significantly worse overall survival and disease-free survival compared to those in the shorter interval group (18). Early intervention can markedly enhance overall survival and disease-free survival rates in poor responders following neoadjuvant chemoradiotherapy, while also mitigating the risk of tumor progression and distant metastasis (18, 19). However, through X-tile analysis, we determined that a cutoff time of 8 weeks resulted in the most favorable overall survival and disease-free survival outcomes for patients with an unfavorable pathological response. Therefore, commencing surgery within a span of eight weeks can optimize survival outcomes by maximizing the efficacy of nCRT while minimizing potential adverse consequences associated with delays.

Total neoadjuvant therapy (TNT) represents an alternative strategy for locally advanced rectal cancer patients, integrating chemoradiotherapy and neoadjuvant chemotherapy prior to surgical intervention (26). Studies such as RAPIDO, PRODIGE-23, and NRG-GI002 have demonstrated that the TNT strategy can enhance the pathological complete response rate, decrease the incidence of distant metastasis, and facilitate organ function preservation (27–29). Therefore, the TNT strategy may achieve satisfactory tumor regression and reduce pre-surgical waiting time, particularly for patients exhibiting an unfavorable pathological response. However, in this study, all patients received standard neoadjuvant chemoradiotherapy rather than TNT therapy, and it remains uncertain whether an 8-week interval is appropriate. And in this study, we found that patients in the longer interval group exhibited significantly worse OS and DFS compared to those in the shorter interval group. Although a similar trend was observed in the TRG 3 group, the difference was not statistically significant for disease-free survival. We determined 8 weeks to be the optimal cut-off value based on overall survival rather than disease-free survival, which is of paramount importance for patients with locally advanced rectal cancer. And the TRG 3 group comprised only 55 patients, with 27 patients in the longer interval subgroup and 28 patients in the shorter interval subgroup. The limited sample size may have contributed to the lack of statistically significant differences. And for these scenarios, the TNT approach may be more appropriate due to enhanced tumor regression and reduced interval time.

The neoadjuvant treatment strategy greatly benefits from the synergistic contribution of chemotherapy and radiotherapy (30). Chemotherapy drugs disrupt cancer cell replication by impacting DNA synthesis, while ionizing radiation directly ionizes atoms within DNA chains or indirectly generates free radicals that damage the structure of DNA (30). These treatments prevent cancer cell multiplication and lead to tumor regression. However, prolonged waiting times may lead to therapy-induced pelvic fibrosis and anatomical difficulties, resulting in a higher conversion rate and longer operative time. Furthermore, a worse mesorectum is associated with an increased risk of local recurrence and medical complications (7, 8). Patients demonstrating a favorable pathological response are more likely to experience tumor regression or even achieve clinical complete response, leading them to consider a “watch-and-wait” strategy as an alternative to surgery. However, for patients with an unfavorable pathological response, delayed surgery not only fails to improve survival but also increases surgical complications due to the adverse effects of radiotherapy (8). Hence, if rectal cancer patients do not achieve a favorable pathological response, it is advisable to reduce the waiting time and proceed with early surgery.

Delaying surgery for patients with an unfavorable pathological response can have negative implications for their survival. Therefore, accurate assessment of the pathological response is crucial in determining the appropriate course of treatment. Magnetic resonance imaging (MRI) plays a significant role in this process, providing valuable diagnostic information to evaluate tumor response to neoadjuvant chemoradiotherapy (31–33). The application of MRI enables accurate determination of T and N stages, and downstaging T and N stages were significantly associated with improved long-term survival in patients with locally advanced rectal cancer following neoadjuvant chemoradiotherapy (31, 34–36). Successful reduction or elimination of the primary tumor enhances the likelihood of successful surgical resection while decreasing the risk of local recurrence. Similarly, mitigating lymph node involvement can prevent regional disease spread, thereby improving overall prognosis. By leveraging the non-invasive and radiation-free attributes of MRI, we can effectively monitor tumor regression or progression, enabling informed decisions regarding optimal timing for surgical intervention. This study demonstrates that patients with an unfavorable pathological response who undergo surgical intervention within 8 weeks after neoadjuvant therapy experience improved survival outcomes. Therefore, it is recommended to perform MRI examination within 8 weeks after neoadjuvant therapy in order to identify patients with an unfavorable pathological response and enable surgical intervention early (37, 38). And radiomics models derived from pre-treatment MRI images can also predict pathological responses and tumor survival, thereby facilitating the earlier identification of patients with poor responses (39). Therefore, MRI images can offer multi-dimensional information to differentiate the pathological responses among patients.

The management of recurrent locally advanced rectal cancer is crucial for patients with poor pathological response. Early assessment and precise treatment are vital for improving the prognosis of these patients. Recurrence diagnosis depends on techniques like electronic colonoscopy and imaging assessments, which help determine the location, size, and presence of distant metastasis (40). Enhanced CT and MRI have accuracy rates of 70.8% and 68.7%, respectively, in detecting locally recurrent rectal cancer that involves adjacent organs. Distant metastasis staging is typically performed using whole-body enhanced CT scans. For lesions of uncertain nature, PET-CT can further confirm the diagnosis. Surgical resection is the preferred treatment for patients with local recurrence (2).For patients who are inoperable or have distant metastasis, treatment plans should be developed within a multidisciplinary team (MDT) framework, considering factors like the patient’s genetic mutations and microsatellite instability to select the most suitable treatment strategy (2, 41).

The study encountered certain limitations. First, this investigation is retrospective in nature and is based solely on the experience of a single institution, which may introduce potential bias and confounding variables. Second, the limited follow-up time of patients is attributed to the delayed development of the TRG scoring system in our hospital. We will continue to conduct comprehensive monitoring and surveillance on patient survival in subsequent periods to generate more detailed data.

Third, postoperative adjuvant therapy plays an important role in influencing patient prognosis. However, due to the absence of detailed data on postoperative adjuvant therapy, this study could not further investigate its potential impact on the conclusions. Future studies will aim to collect and analyze this data to explore its impact.

## 5 Conclusions

In conclusion, the implementation of a robust surveillance and monitoring system following neoadjuvant chemoradiotherapy is crucial for prompt identification of patients with an unfavorable pathological response. This proactive approach ensures that individuals who would benefit from timely radical surgery within 8 weeks receive optimal care while minimizing potential risks associated with delayed interventions.

## Data Availability

The raw data supporting the conclusions of this article will be made available by the authors, without undue reservation.
