# Supplementary material for: The optimal time interval between neoadjuvant chemoradiotherapy and surgery for patients with an unfavorable pathological response in locally advanced rectal cancer: a retrospective cohort study
Source: Front Oncol. 2025 Feb 14;15:1534148. doi: 10.3389/fonc.2025.1534148 (PMC11867938; doi:10.3389/fonc.2025.1534148)
Supplement: Supplementary file 1 [file Presentation1.pdf]

Supplementary Material

1 Supplementary Figures

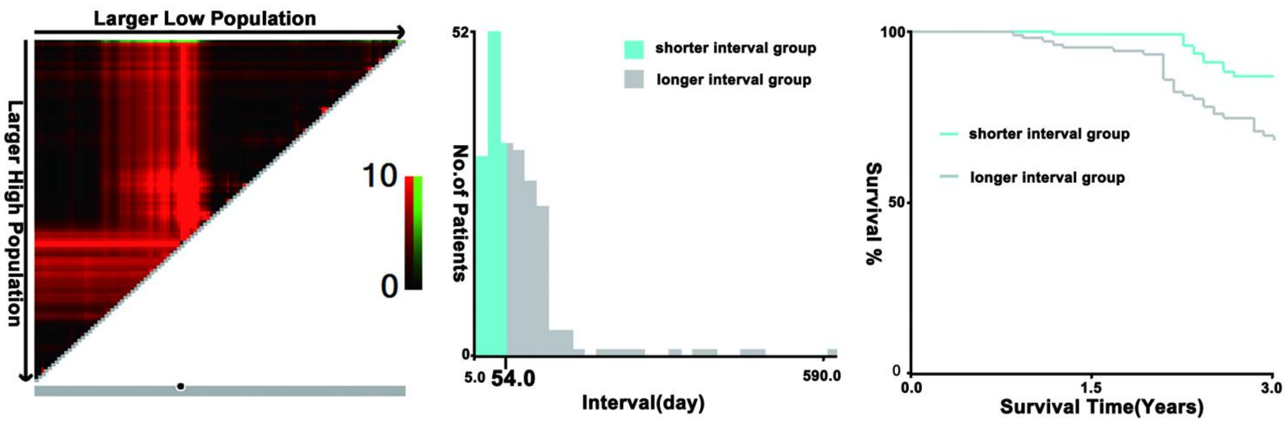

Supplementary Figure 1. The determination of the optimal cut-off value for the time interval between neoadjuvant chemoradiotherapy and surgery using X-tile analysis. The cursor can be manually moved to any colored area of the chart (A) to select the best cut-off point (B) and to display the survival curve (C). The optimal cut-off value was 54 days, and the samples were divided into shorter interval group and longer interval group. The Kaplan-Meier graph illustrates the OS after stratifying the groups based on optimal truncation values.

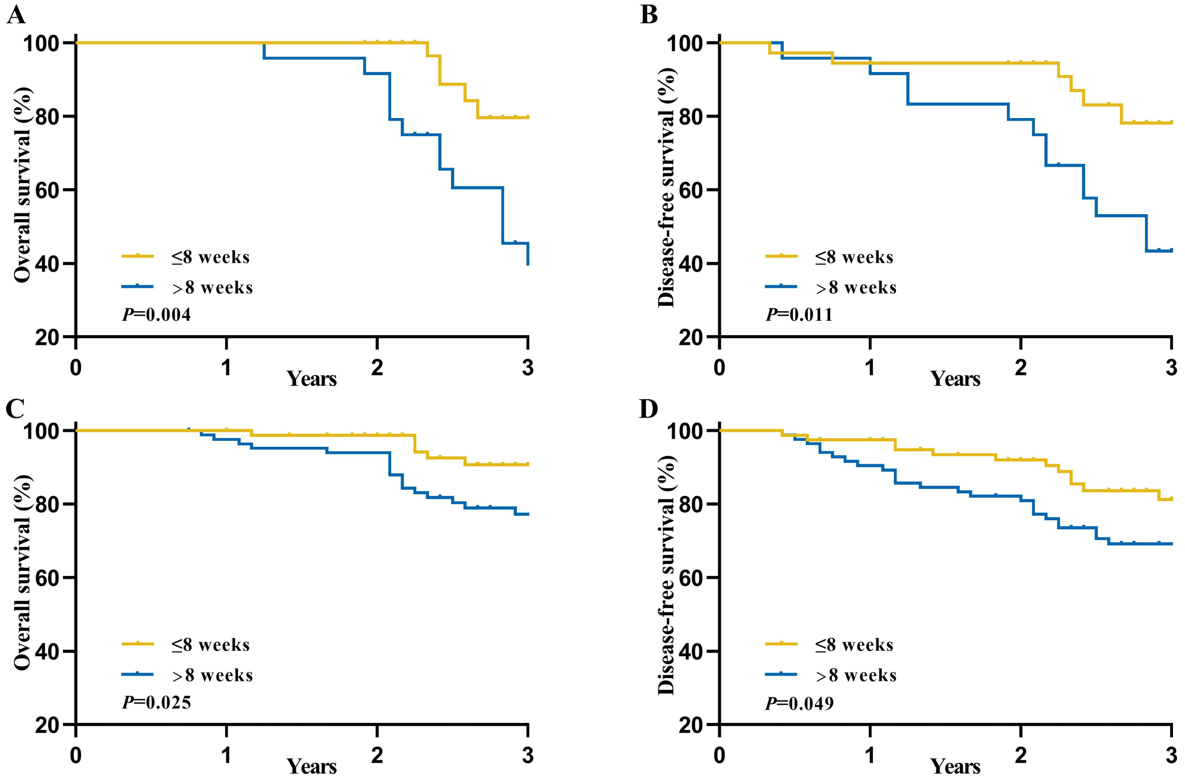

Supplementary fig. 2.

Long-term survival of patients with an unfavorable pathological response in locally advanced rectal cancer, comparing the longer interval and shorter interval groups according to chemotherapy regimens. (A) Overall survival for the capecitabine group; (B) Disease-free survival for the capecitabine group; (C) Overall survival for the CapeOx group; (D) Disease-free survival for the CapeOx group.

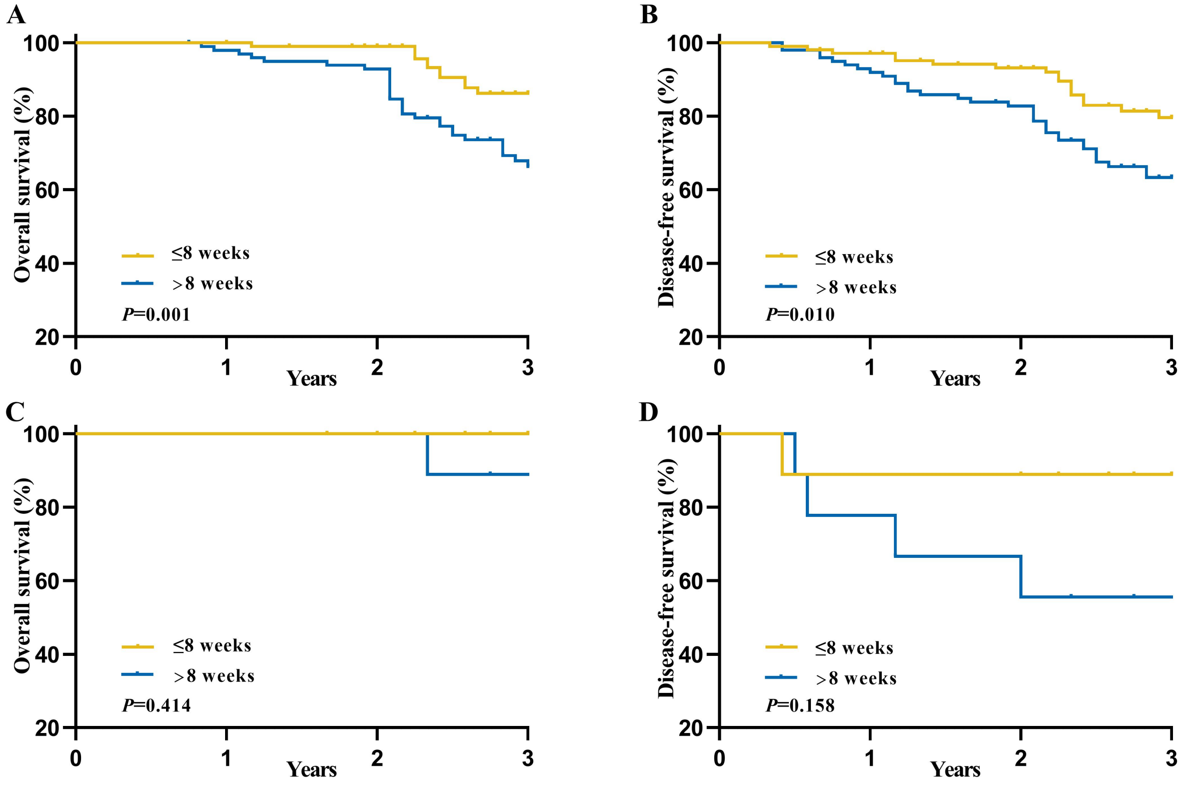

Supplementary Fig. 3.
Long-term survival of patients with an unfavorable pathological response in locally advanced rectal cancer, comparing the longer interval and shorter interval groups according to radiotherapy regimens. (A) Overall survival for the long-term radiotherapy group; (B) Disease-free survival for the long-term radiotherapy group; (C) Overall survival for the short-term radiotherapy group; (D) Disease-free survival for the short-term radiotherapy group.

2 Supplementary Table

Supplementary table 1. Comparison of median follow-up duration .

**Characteristic**	**Total(n,%)**	**Interval**<br>**Follow-up Time,M(Q1,Q3)**	***p***
**All patients**	222	34.0(28.0,42.0)	
**Interval**			0.371
≤8 weeks	114 (51.35)	33.0 (28.0,40.8)	
＞8 weeks	108 (48.65)	34.5 (27.8,44.0)	
